# 5,15-Bis(4-pentyl­oxyphen­yl)porphyrin

**DOI:** 10.1107/S160053681301550X

**Published:** 2013-06-08

**Authors:** Mathias O. Senge

**Affiliations:** a School of Chemistry, SFI Tetrapyrrole Laboratory, Trinity Biomedical Sciences Institute, 152-160 Pearse Street, Trinity College Dublin, Dublin 2, Ireland

## Abstract

In the title compound, C_42_H_42_N_4_O_2_, the complete molecule is generated by a crystallographic inversion centre. The porphyrin system exhibits a near planar macrocycle conformation with an average deviation from the least-squares plane of the 24 macrocycle atoms of 0.037 (5) Å. The phenyl *ipso* C atoms are positioned above and below the porphyrin plane by 0.35 (1) Å and the macrocycle shows evidence of in-plane rectangular elongation with N⋯N separations of 3.032 (5) and 2.803 (5) Å. Two intramolecular N—H⋯N hydrogen bonds occur.

## Related literature

For the conformation of porphyrins, see: Scheidt & Lee (1987 ▶); Senge *et al.* (1997 ▶); Senge (2006 ▶). For the synthesis of such compounds, see: Wiehe *et al.* (2005 ▶).
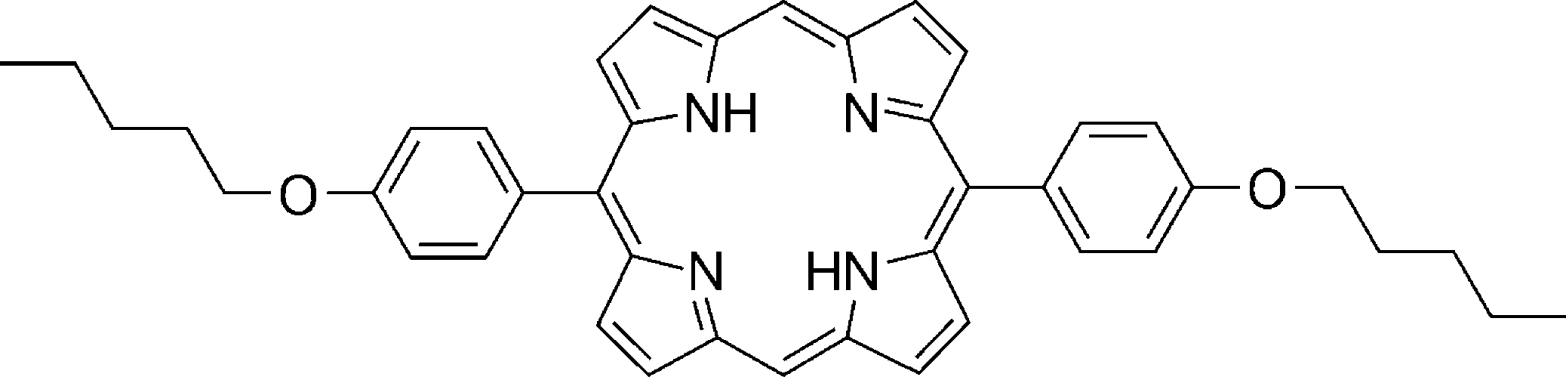


## Experimental

### 

#### Crystal data

C_42_H_42_N_4_O_2_
*M* *_r_* = 634.80
Triclinic, 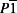

*a* = 9.5222 (6) Å
*b* = 9.5799 (6) Å
*c* = 10.2195 (6) Å
α = 67.777 (1)°
β = 88.063 (1)°
γ = 72.464 (1)°
*V* = 819.49 (9) Å^3^
*Z* = 1
Mo *K*α radiation
μ = 0.08 mm^−1^
*T* = 90 K
0.30 × 0.10 × 0.08 mm

#### Data collection

Bruker SMART APEXII diffractometer
Absorption correction: multi-scan (*SADABS*; Bruker, 2005 ▶) *T*_min_ = 0.97, *T*_max_ = 0.99
9093 measured reflections
3606 independent reflections
2489 reflections with *I* > 2σ(*I*)
*R*_int_ = 0.039

#### Refinement

*R*[*F* ^2^ > 2σ(*F* ^2^)] = 0.045
*wR*(*F* ^2^) = 0.109
*S* = 1.04
3606 reflections
219 parameters
H-atom parameters constrained
Δρ_max_ = 0.27 e Å^−3^
Δρ_min_ = −0.23 e Å^−3^

### 

Data collection: *APEX2* (Bruker, 2005 ▶); cell refinement: *SAINT* (Bruker, 2005 ▶); data reduction: *SAINT*; program(s) used to solve structure: *SHELXS97* (Sheldrick, 2008 ▶); program(s) used to refine structure: *SHELXL97* (Sheldrick, 2008 ▶); molecular graphics: *XP* in *SHELXTL* (Sheldrick, 2008 ▶); software used to prepare material for publication: *XP* in *SHELXTL*.

## Supplementary Material

Crystal structure: contains datablock(s) I, 2R. DOI: 10.1107/S160053681301550X/zl2552sup1.cif

Structure factors: contains datablock(s) I. DOI: 10.1107/S160053681301550X/zl2552Isup2.hkl

Additional supplementary materials:  crystallographic information; 3D view; checkCIF report

## Figures and Tables

**Table 1:** Hydrogen-bond geometry (Å, °)

*D*—H⋯*A*	*D*—H	H⋯*A*	*D*⋯*A*	*D*—H⋯*A*
N21—H21⋯N24	0.88	2.50	3.033 (2)	119
N21—H21⋯N24^i^	0.88	2.22	2.804 (2)	123

Symmetry code: (i) 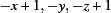.

**Fig. 1.**
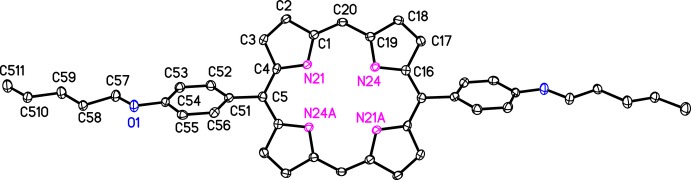
: Molecular structure of the title compound. Thermal ellipsoids are drawn at 50% probability level; hydrogen atoms have been omitted for clarity.

## supplementary crystallographic information

### Comment

Many porphyrin structures with four *meso* substituents have been reported (Scheidt & Lee, 1987). The available number of structures for systems with only two *meso* residues is much smaller. In the context of an ongoing program on the conformational flexibilty of porphyrins (Senge, 2006) we are interested in a comparative analysis of 5,10-A_2_– and 5,15-A_2_-disubstituted porphyrins. The title compound is an example for the latter and exhibits a planar macrocycle with an average deviation from the least-squares-plane of the 24 macrocycle atoms of Δ24 = 0.037 (5) Å. The phenyl *ipso* carbon atoms are positioned above and below the porphyrin plane by 0.35 Å and the macrocycle shows evidence for in-plane distortion with N···N separations of 3.032 (5) and 2.803 (5) Å. This is similar to the situation found in 2,3,5,7,8,12,13,15, 17,18- decasubstituted porphyrins (Senge *et al.*, 1997) where *peri* interaction between the *meso* and beta substituents occur. The molecules pack in parallel layers with the alkyl chains separating the macrocycles and only minimal π-aggregation.

### Experimental

The compound was prepared as described by Wiehe *et al.* (2005) and crystallized from CH_2_Cl_2_/CH_3_OH.

### Refinement

All nonhydrogen atoms were refined with anisotropic thermal parameters. Hydrogen atoms were refined with a standard riding model (C—H distance 0.96 - 0.99 Å, *U*_iso_ = 1.2–1.5 times of parent atom). Pyrrole hydrogen atoms were located in difference maps and refined with isotropic thermal parameters.

### Crystal data

**Table:**

C_42_H_42_N_4_O_2_	*Z* = 1
*M**_r_* = 634.80	*F*(000) = 338
Triclinic, *P*1	*D*_x_ = 1.286 Mg m^−^^3^*D*_m_ = n/d Mg m^−^^3^*D*_m_ measured by not measured
Hall symbol: -P 1	Melting point: n/d K
*a* = 9.5222 (6) Å	Mo *K*α radiation, λ = 0.71073 Å
*b* = 9.5799 (6) Å	Cell parameters from 1771 reflections
*c* = 10.2195 (6) Å	θ = 4.5–60.7°
α = 67.777 (1)°	µ = 0.08 mm^−^^1^
β = 88.063 (1)°	*T* = 90 K
γ = 72.464 (1)°	Parallelpiped, red
*V* = 819.49 (9) Å^3^	0.30 × 0.10 × 0.08 mm

### Data collection

**Table:**

Bruker SMART APEXII diffractometer	3606 independent reflections
Radiation source: fine-focus sealed tube	2489 reflections with *I* > 2σ(*I*)
Graphite monochromator	*R*_int_ = 0.039
Detector resolution: 8.3 pixels mm^-1^	θ_max_ = 27.1°, θ_min_ = 2.2°
ω scans	*h* = −12→12
Absorption correction: multi-scan (*SADABS*; Bruker, 2005)	*k* = −12→12
*T*_min_ = 0.97, *T*_max_ = 0.99	*l* = −13→13
9093 measured reflections	

### Refinement

**Table:**

Refinement on *F*^2^	Primary atom site location: structure-invariant direct methods
Least-squares matrix: full	Secondary atom site location: difference Fourier map
*R*[*F*^2^ > 2σ(*F*^2^)] = 0.045	Hydrogen site location: inferred from neighbouring sites
*wR*(*F*^2^) = 0.109	H-atom parameters constrained
*S* = 1.04	*w* = 1/[σ^2^(*F*_o_^2^) + (0.0433*P*)^2^ + 0.1316*P*] where *P* = (*F*_o_^2^ + 2*F*_c_^2^)/3
3606 reflections	(Δ/σ)_max_ < 0.001
219 parameters	Δρ_max_ = 0.27 e Å^−^^3^
0 restraints	Δρ_min_ = −0.23 e Å^−^^3^

### Special details

**Table:**

Geometry. All e.s.d.'s (except the e.s.d. in the dihedral angle between two l.s. planes) are estimated using the full covariance matrix. The cell e.s.d.'s are taken into account individually in the estimation of e.s.d.'s in distances, angles and torsion angles; correlations between e.s.d.'s in cell parameters are only used when they are defined by crystal symmetry. An approximate (isotropic) treatment of cell e.s.d.'s is used for estimating e.s.d.'s involving l.s. planes.
Refinement. Refinement of *F*^2^ against ALL reflections. The weighted *R*-factor *wR* and goodness of fit *S* are based on *F*^2^, conventional *R*-factors *R* are based on *F*, with *F* set to zero for negative *F*^2^. The threshold expression of *F*^2^ > σ(*F*^2^) is used only for calculating *R*-factors(gt) *etc*. and is not relevant to the choice of reflections for refinement. *R*-factors based on *F*^2^ are statistically about twice as large as those based on *F*, and *R*- factors based on ALL data will be even larger.

### Fractional atomic coordinates and isotropic or equivalent isotropic displacement parameters (Å^2^)

**Table:**

	*x*	*y*	*z*	*U*_iso_*/*U*_eq_	
N21	0.64309 (14)	−0.19503 (16)	0.46008 (14)	0.0171 (3)	
H21	0.5815	−0.1069	0.4627	0.047 (6)*	
N24	0.36841 (13)	−0.12143 (15)	0.61217 (14)	0.0160 (3)	
C1	0.62390 (16)	−0.34160 (19)	0.52069 (17)	0.0179 (4)	
C2	0.74472 (17)	−0.4492 (2)	0.48647 (18)	0.0190 (4)	
H2A	0.7600	−0.5592	0.5123	0.023*	
C3	0.83431 (17)	−0.36651 (19)	0.41037 (17)	0.0185 (4)	
H3A	0.9229	−0.4090	0.3740	0.022*	
C4	0.77200 (16)	−0.20511 (19)	0.39479 (17)	0.0169 (4)	
C5	0.83034 (16)	−0.07952 (19)	0.33369 (17)	0.0169 (4)	
C16	0.24129 (16)	−0.07563 (19)	0.67302 (16)	0.0164 (3)	
C17	0.18961 (17)	−0.2107 (2)	0.74985 (18)	0.0195 (4)	
H17A	0.1041	−0.2097	0.8006	0.023*	
C18	0.28721 (17)	−0.3376 (2)	0.73457 (18)	0.0198 (4)	
H18A	0.2843	−0.4437	0.7732	0.024*	
C19	0.39727 (16)	−0.28065 (19)	0.64781 (17)	0.0172 (4)	
C20	0.51314 (16)	−0.37959 (19)	0.60556 (17)	0.0185 (4)	
H20A	0.5167	−0.4877	0.6396	0.022*	
C51	0.98127 (17)	−0.11283 (19)	0.28262 (17)	0.0177 (4)	
C52	1.01657 (17)	−0.1671 (2)	0.17315 (18)	0.0206 (4)	
H52A	0.9421	−0.1859	0.1286	0.025*	
C53	1.15769 (17)	−0.1945 (2)	0.12745 (18)	0.0207 (4)	
H53A	1.1792	−0.2316	0.0527	0.025*	
C54	1.26742 (16)	−0.16720 (19)	0.19238 (18)	0.0188 (4)	
C55	1.23542 (17)	−0.11454 (19)	0.30202 (17)	0.0192 (4)	
H55A	1.3103	−0.0965	0.3467	0.023*	
C56	1.09475 (17)	−0.08822 (19)	0.34664 (18)	0.0192 (4)	
H56A	1.0744	−0.0526	0.4224	0.023*	
O1	1.41004 (11)	−0.18754 (14)	0.15628 (12)	0.0226 (3)	
C57	1.45008 (17)	−0.2298 (2)	0.03660 (18)	0.0227 (4)	
H57A	1.3832	−0.1524	−0.0488	0.027*	
H57B	1.4430	−0.3366	0.0554	0.027*	
C58	1.60744 (17)	−0.2284 (2)	0.01421 (18)	0.0211 (4)	
H58A	1.6702	−0.2943	0.1048	0.025*	
H58B	1.6106	−0.1185	−0.0147	0.025*	
C59	1.66924 (17)	−0.2919 (2)	−0.09922 (19)	0.0249 (4)	
H59A	1.6492	−0.3934	−0.0788	0.030*	
H59B	1.6167	−0.2157	−0.1927	0.030*	
C510	1.83383 (17)	−0.3191 (2)	−0.10777 (18)	0.0217 (4)	
H51A	1.8868	−0.3995	−0.0159	0.026*	
H51B	1.8546	−0.2190	−0.1236	0.026*	
C511	1.89275 (18)	−0.3744 (2)	−0.22522 (19)	0.0277 (4)	
H51C	2.0009	−0.4029	−0.2182	0.042*	
H51D	1.8518	−0.2889	−0.3175	0.042*	
H51E	1.8637	−0.4673	−0.2159	0.042*	

### Atomic displacement parameters (Å^2^)

**Table:**

	*U*^11^	*U*^22^	*U*^33^	*U*^12^	*U*^13^	*U*^23^
N21	0.0139 (7)	0.0134 (7)	0.0229 (8)	−0.0023 (5)	0.0014 (5)	−0.0075 (6)
N24	0.0136 (6)	0.0145 (7)	0.0195 (7)	−0.0030 (5)	−0.0003 (5)	−0.0071 (6)
C1	0.0159 (8)	0.0165 (8)	0.0220 (9)	−0.0040 (6)	−0.0007 (6)	−0.0088 (7)
C2	0.0184 (8)	0.0141 (8)	0.0241 (9)	−0.0023 (6)	0.0006 (7)	−0.0090 (7)
C3	0.0148 (8)	0.0197 (9)	0.0206 (9)	−0.0017 (6)	0.0021 (6)	−0.0103 (7)
C4	0.0119 (7)	0.0192 (9)	0.0184 (9)	−0.0012 (6)	0.0004 (6)	−0.0087 (7)
C5	0.0151 (8)	0.0190 (9)	0.0171 (8)	−0.0047 (6)	0.0008 (6)	−0.0078 (7)
C16	0.0156 (8)	0.0186 (9)	0.0146 (8)	−0.0046 (6)	0.0001 (6)	−0.0065 (7)
C17	0.0179 (8)	0.0206 (9)	0.0199 (9)	−0.0071 (7)	0.0040 (6)	−0.0071 (7)
C18	0.0193 (8)	0.0156 (9)	0.0237 (9)	−0.0066 (7)	0.0022 (7)	−0.0059 (7)
C19	0.0153 (8)	0.0159 (8)	0.0201 (9)	−0.0046 (6)	−0.0004 (6)	−0.0066 (7)
C20	0.0180 (8)	0.0136 (8)	0.0236 (9)	−0.0054 (6)	−0.0001 (6)	−0.0063 (7)
C51	0.0171 (8)	0.0144 (8)	0.0191 (9)	−0.0031 (6)	0.0028 (6)	−0.0052 (7)
C52	0.0183 (8)	0.0214 (9)	0.0221 (9)	−0.0066 (7)	0.0012 (7)	−0.0080 (8)
C53	0.0232 (9)	0.0212 (9)	0.0189 (9)	−0.0060 (7)	0.0045 (7)	−0.0100 (7)
C54	0.0146 (8)	0.0146 (8)	0.0222 (9)	−0.0026 (6)	0.0047 (6)	−0.0036 (7)
C55	0.0193 (8)	0.0165 (9)	0.0204 (9)	−0.0057 (7)	−0.0015 (7)	−0.0053 (7)
C56	0.0197 (8)	0.0182 (9)	0.0184 (9)	−0.0034 (7)	0.0021 (6)	−0.0078 (7)
O1	0.0168 (6)	0.0286 (7)	0.0251 (7)	−0.0071 (5)	0.0065 (5)	−0.0135 (6)
C57	0.0202 (8)	0.0268 (10)	0.0229 (9)	−0.0062 (7)	0.0069 (7)	−0.0127 (8)
C58	0.0180 (8)	0.0183 (9)	0.0233 (9)	−0.0047 (7)	0.0060 (7)	−0.0050 (7)
C59	0.0204 (9)	0.0312 (11)	0.0260 (10)	−0.0096 (8)	0.0071 (7)	−0.0135 (8)
C510	0.0185 (8)	0.0216 (9)	0.0237 (9)	−0.0046 (7)	0.0049 (7)	−0.0088 (8)
C511	0.0209 (9)	0.0360 (11)	0.0324 (11)	−0.0105 (8)	0.0071 (8)	−0.0190 (9)

### Geometric parameters (Å, º)

**Table:**

N21—C1	1.370 (2)	C52—H52A	0.9500
N21—C4	1.3722 (19)	C53—C54	1.394 (2)
N21—H21	0.8800	C53—H53A	0.9500
N24—C19	1.367 (2)	C54—O1	1.3710 (18)
N24—C16	1.3711 (19)	C54—C55	1.384 (2)
C1—C20	1.388 (2)	C55—C56	1.381 (2)
C1—C2	1.428 (2)	C55—H55A	0.9500
C2—C3	1.362 (2)	C56—H56A	0.9500
C2—H2A	0.9500	O1—C57	1.4335 (19)
C3—C4	1.427 (2)	C57—C58	1.511 (2)
C3—H3A	0.9500	C57—H57A	0.9900
C4—C5	1.399 (2)	C57—H57B	0.9900
C5—C16^i^	1.412 (2)	C58—C59	1.527 (2)
C5—C51	1.496 (2)	C58—H58A	0.9900
C16—C5^i^	1.412 (2)	C58—H58B	0.9900
C16—C17	1.457 (2)	C59—C510	1.515 (2)
C17—C18	1.348 (2)	C59—H59A	0.9900
C17—H17A	0.9500	C59—H59B	0.9900
C18—C19	1.448 (2)	C510—C511	1.514 (2)
C18—H18A	0.9500	C510—H51A	0.9900
C19—C20	1.396 (2)	C510—H51B	0.9900
C20—H20A	0.9500	C511—H51C	0.9800
C51—C52	1.394 (2)	C511—H51D	0.9800
C51—C56	1.403 (2)	C511—H51E	0.9800
C52—C53	1.389 (2)		
			
C1—N21—C4	110.37 (13)	C54—C53—H53A	120.3
C1—N21—H21	124.8	O1—C54—C55	114.91 (14)
C4—N21—H21	124.8	O1—C54—C53	125.17 (15)
C19—N24—C16	105.16 (13)	C55—C54—C53	119.92 (14)
N21—C1—C20	126.59 (15)	C56—C55—C54	120.09 (15)
N21—C1—C2	106.65 (13)	C56—C55—H55A	120.0
C20—C1—C2	126.68 (15)	C54—C55—H55A	120.0
C3—C2—C1	108.13 (15)	C55—C56—C51	121.49 (16)
C3—C2—H2A	125.9	C55—C56—H56A	119.3
C1—C2—H2A	125.9	C51—C56—H56A	119.3
C2—C3—C4	108.17 (14)	C54—O1—C57	118.76 (12)
C2—C3—H3A	125.9	O1—C57—C58	106.88 (13)
C4—C3—H3A	125.9	O1—C57—H57A	110.3
N21—C4—C5	124.67 (14)	C58—C57—H57A	110.3
N21—C4—C3	106.62 (14)	O1—C57—H57B	110.3
C5—C4—C3	128.61 (14)	C58—C57—H57B	110.3
C4—C5—C16^i^	123.29 (14)	H57A—C57—H57B	108.6
C4—C5—C51	118.72 (14)	C57—C58—C59	111.63 (14)
C16^i^—C5—C51	117.82 (14)	C57—C58—H58A	109.3
N24—C16—C5^i^	125.69 (15)	C59—C58—H58A	109.3
N24—C16—C17	110.71 (14)	C57—C58—H58B	109.3
C5^i^—C16—C17	123.59 (14)	C59—C58—H58B	109.3
C18—C17—C16	106.36 (14)	H58A—C58—H58B	108.0
C18—C17—H17A	126.8	C510—C59—C58	113.42 (14)
C16—C17—H17A	126.8	C510—C59—H59A	108.9
C17—C18—C19	106.69 (15)	C58—C59—H59A	108.9
C17—C18—H18A	126.7	C510—C59—H59B	108.9
C19—C18—H18A	126.7	C58—C59—H59B	108.9
N24—C19—C20	126.46 (14)	H59A—C59—H59B	107.7
N24—C19—C18	111.07 (13)	C59—C510—C511	113.04 (14)
C20—C19—C18	122.44 (15)	C59—C510—H51A	109.0
C1—C20—C19	128.88 (15)	C511—C510—H51A	109.0
C1—C20—H20A	115.6	C59—C510—H51B	109.0
C19—C20—H20A	115.6	C511—C510—H51B	109.0
C52—C51—C56	117.34 (14)	H51A—C510—H51B	107.8
C52—C51—C5	123.54 (14)	C510—C511—H51C	109.5
C56—C51—C5	119.12 (15)	C510—C511—H51D	109.5
C53—C52—C51	121.78 (15)	H51C—C511—H51D	109.5
C53—C52—H52A	119.1	C510—C511—H51E	109.5
C51—C52—H52A	119.1	H51C—C511—H51E	109.5
C52—C53—C54	119.38 (16)	H51D—C511—H51E	109.5
C52—C53—H53A	120.3		
			
C4—N21—C1—C20	174.18 (15)	C2—C1—C20—C19	176.51 (16)
C4—N21—C1—C2	−2.51 (18)	N24—C19—C20—C1	0.4 (3)
N21—C1—C2—C3	1.54 (18)	C18—C19—C20—C1	178.43 (16)
C20—C1—C2—C3	−175.14 (16)	C4—C5—C51—C52	63.4 (2)
C1—C2—C3—C4	−0.05 (19)	C16^i^—C5—C51—C52	−121.15 (18)
C1—N21—C4—C5	−174.17 (15)	C4—C5—C51—C56	−116.84 (18)
C1—N21—C4—C3	2.48 (18)	C16^i^—C5—C51—C56	58.6 (2)
C2—C3—C4—N21	−1.45 (18)	C56—C51—C52—C53	−0.7 (2)
C2—C3—C4—C5	175.01 (16)	C5—C51—C52—C53	179.07 (15)
N21—C4—C5—C16^i^	−2.8 (3)	C51—C52—C53—C54	0.0 (2)
C3—C4—C5—C16^i^	−178.73 (16)	C52—C53—C54—O1	−179.13 (15)
N21—C4—C5—C51	172.32 (14)	C52—C53—C54—C55	0.5 (2)
C3—C4—C5—C51	−3.6 (3)	O1—C54—C55—C56	179.34 (14)
C19—N24—C16—C5^i^	179.15 (15)	C53—C54—C55—C56	−0.4 (2)
C19—N24—C16—C17	0.37 (17)	C54—C55—C56—C51	−0.4 (2)
N24—C16—C17—C18	0.14 (18)	C52—C51—C56—C55	0.9 (2)
C5^i^—C16—C17—C18	−178.68 (15)	C5—C51—C56—C55	−178.90 (15)
C16—C17—C18—C19	−0.56 (18)	C55—C54—O1—C57	−175.16 (14)
C16—N24—C19—C20	177.47 (16)	C53—C54—O1—C57	4.5 (2)
C16—N24—C19—C18	−0.72 (17)	C54—O1—C57—C58	174.70 (13)
C17—C18—C19—N24	0.84 (19)	O1—C57—C58—C59	172.84 (13)
C17—C18—C19—C20	−177.45 (15)	C57—C58—C59—C510	−170.19 (15)
N21—C1—C20—C19	0.5 (3)	C58—C59—C510—C511	−177.21 (15)

Symmetry code: (i) −*x*+1, −*y*, −*z*+1.

### Hydrogen-bond geometry (Å, º)

**Table:**

*D*—H···*A*	*D*—H	H···*A*	*D*···*A*	*D*—H···*A*
N21—H21···N24	0.88	2.50	3.033 (2)	119
N21—H21···N24^i^	0.88	2.22	2.804 (2)	123

Symmetry code: (i) −*x*+1, −*y*, −*z*+1.
